# Acute Changes in Mentation in a Patient with Hepatic Cirrhosis Treated with High Doses of Dexamethasone

**DOI:** 10.7759/cureus.1675

**Published:** 2017-09-10

**Authors:** Luis Dabul, Andrew Droney, Juan Oms, Marcos A Sanchez-Gonzalez

**Affiliations:** 1 Department of Psychiatry, Larkin Community Hospital; 2 Student, Lecom-bradenton; 3 Department of Psychiatry, Larking Community Hospital; 4 Division of Clinical & Translational Research, Larkin Community Hospital

**Keywords:** steroid induced delirium, hepatic encephalopathy

## Abstract

Despite the anti-inflammatory benefits of steroids in the management of multiple medical conditions, they are associated with undesired metabolic and psychiatric side effects. We present a case of a 57-year-old Hispanic man with hepatic cirrhosis due to hepatitis C and no past medical history of psychiatric illnesses who became delirious after treatment with high doses of intravenous Dexamethasone. The patient presented to Larkin Community Hospital, USA with complaints of lower back pain requiring treatment with steroids for severe lumbar central canal stenosis. After three days of treatment, the patient became disoriented to time and place, grossly psychotic with auditory hallucinations and disorganized behavior, manic, aggressive, combative, restless, hard to redirect, and unable to follow commands. He met the criteria for a diagnosis of substance-induced psychotic disorder according to Diagnostic and Statistical Manual of Mental Disorders (DSM) V. Furthermore, the patient had worsening hepatic profile, a high ammonia level of 125 umol/L, and clinical findings consistent with West Haven classification grade 2 encephalopathy. Head computed tomography (CT) scan was normal. He was treated with discontinuation of steroids, lactulose, and Haloperidol returning to baseline mental status after 48 hours. The patient's hospitalization was complicated with a prolonged hospital stay after lumbar surgery. This case illustrates that treatment with high doses of Dexamethasone in a patient with hepatic cirrhosis can cause acute changes in mental status by (i) inducing delirium, and (ii) precipitating hepatic encephalopathy.

## Introduction

High dose oral corticosteroid therapy increases the risk of psychiatric events such as mood disorders, anxiety, aggressive behavior, insomnia, agitation, depersonalization, panic disorder, suicidal thinking, dementia, and delirium [1]. Thus, the term "steroid-induced psychosis" does not describe these adverse events. Furthermore, the incidence of adverse psychiatric symptoms has a direct relationship with the dose of oral corticosteroids [2]. Nonetheless, there are a few reported cases of psychosis following the administration of low dose of corticosteroids. Psychosis has been reported to occur as early as during the first week of steroid therapy, with the resolution of symptoms within a week after cessation of therapy [3].

The differential diagnoses of acute changes in mentation are very broad and should include hepatic encephalopathy (HE) in patients with hepatic cirrhosis. The psychiatric manifestations found in West Haven classification Grade 2 HE can mimic steroid-induced psychosis manifesting as delirium and mania [4]. HE in the clinical setting is evaluated by monitoring serum laboratories of hepatic function, ammonia, and bilirubin. Interestingly, ammonia levels do not always correlate with the severity of HE, and there are numerous contributing factors to its severity such as blood–brain barrier permeability, cytokines, and various toxins [5].

**Figure 1:**
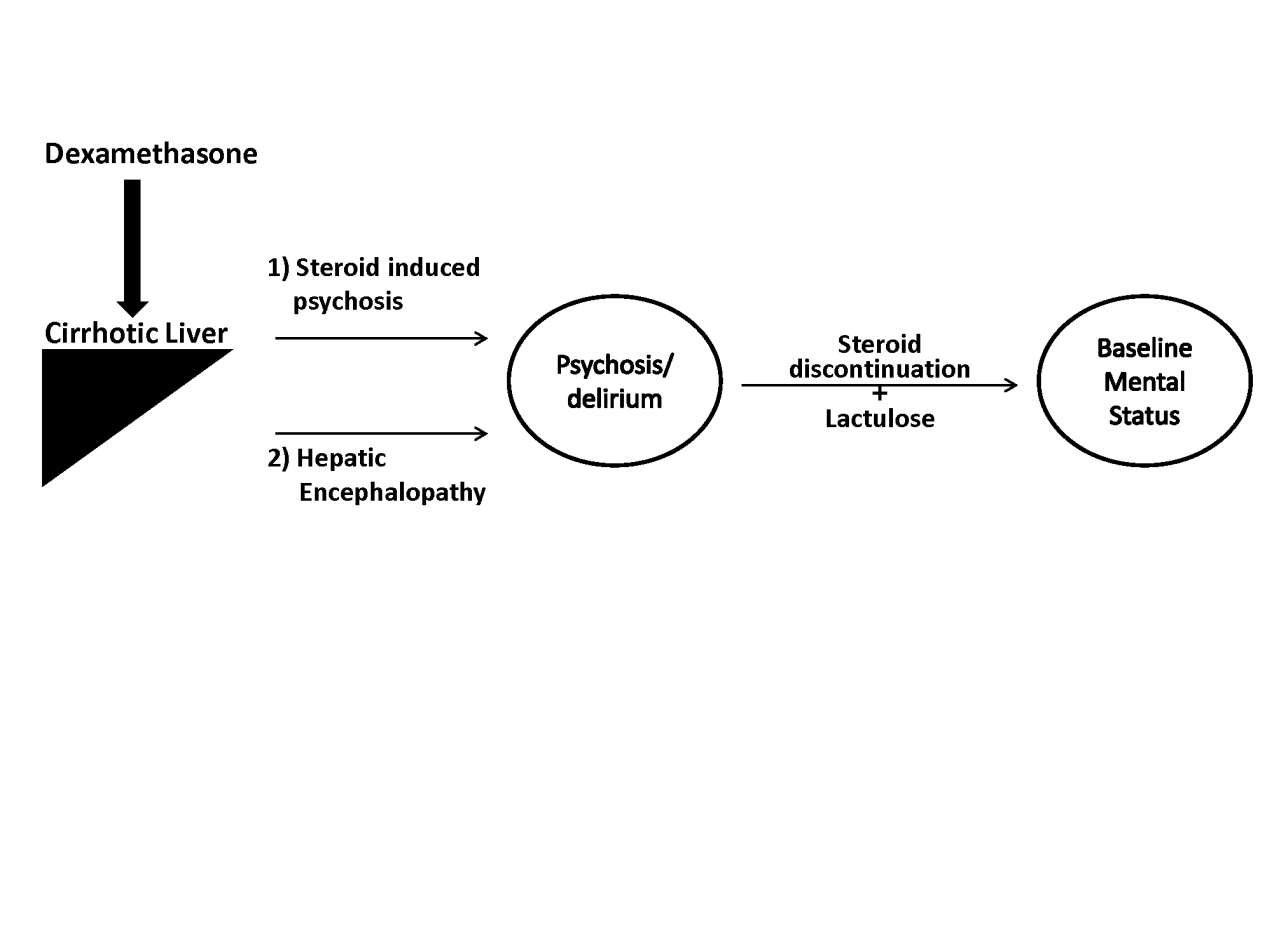
High doses of steroids cause acute changes in mentation in a patient with liver cirrhosis.

This case report illustrates a patient with hepatic cirrhosis due to hepatitis C who developed acute changes in mentation after treatment with high doses of Dexamethasone. A detailed examination of the case revealed that the high doses of steroid therapy i) induced delirium and ii) precipitated HE (Figure 1). The case describes the patient’s history of illness from the development of acute changes in mentation to return to baseline mental status and hospital course after lumbar neurosurgery.

## Case presentation

The patient is a 57-year-old man with no past medical history of psychiatry illnesses who presented to Larkin Community Hospital emergency department with complaints of severe lower back pain. On physical examination, he was calm and cooperative, alert, awake, and oriented to self, place, and time. Chest radiographs showed no abnormal finding. Magnetic resonance imaging (MRI) of the lumbar spine showed he had multilevel degenerative changes most pronounced at L5-S1 with mild, moderate, and severe central canal stenosis caused by broad base disc bulges requiring treatment with high doses of high potency corticosteroid therapy. He received a total of 32 mg/ml of Dexamethasone intravenous in a period of three days spaced into eight doses of 4 mg/ml given every six hours. With the first doses of steroids, his lower back pain improved significantly.

On hospital day 3, he became delirious indicated by acute changes in mentation being disoriented to time and place, with poor attention and concentration, hard to redirect, unable to follow commands, irritable, hyperactive, irrational, incoherent, and psychotic. He was aggressive and combative towards the staff at the hospital and his roommate. Furthermore, he was transferred to a room with a single bed with 24-hour one on one supervision from hospital nursing staff. He experienced auditory hallucinations, was seen speaking to himself and screaming in a loud voice “Shut up, stop talking to me” when no one was around him. His affect was blunted and mood was manic. He was restless, constantly moving from supine to sitting position on the hospital bed. His speech was poor, with fast rate, high volume, disorganized, incoherent, and repetitive. Mental status assessed using the West Haven Criteria was graded at 2. On the Glasgow Coma Scale, he scored 13.

Serum laboratories were significant for thrombocytopenia (Table 1) and elevation of liver enzymes and ammonia (Table 2). Human immunodeficiency virus test was negative. Hepatitis A and B tests were negative, and hepatitis C was positive. Ammonia level was 125.0 umol/L during the psychotic episode and measured after 48 hours with the resolution of psychosis was 37 umol/L (16-53 umol/L). Thyroid-stimulating hormone (TSH) was 1.06 (0.3-4.20 uU/mL), carcinoembryonic antigen (CEA) 2.4 (0.7-5.0 ng/ml), prostate-specific antigen (PSA) 0.56 (0.30-4.0 ng/ml), and uric acid 1.7 (3.4-7.0 mg/dL). Blood tests for clotting time were prolonged (Table 3).

**Table 1:** Blood cells count of the patient at presentation and after delirium. WBC: White blood cell; RBC: Red blood cell; HGB: Hemoglobin; HCT: Hematocrit; MCV: Mean corpuscular volume; MCHC: Mean corpuscular hemoglobin concentration; RDW: Red blood cell distribution width.

Blood Cell Count	Initial Presentation	After Delirium
WBC (10^3^/mcL)	4.7	6.5
RBC (10^6^/mcL)	3.72	3.54
HGB (g/dl)	12.1	11.8
HCT (%)	34	33
MCV (fL)	92.3	92.9
MCHC (g/dl)	35.3	35.8
RDW (%)	12.2	13.0
Platelets (10^3^/mcL)	56	52
% Neutrophils	62.8	78.3
% Lymphocytes	24.0	10.6
% Monocytes	10.2	11.0
% Eosinophils	2.0	0.0
% Basophils	1.0	0.2
# Absolute Neutrophils (10^3^/mcL)	2.9	5.1
# Absolute Lymphocytes (10^3^/mcL)	1.1	0.7
# Absolute Monocytes (10^3^/mcL)	0.5	0.7
# Absolute Eosinophils (10^3^/mcL)	0.1	0.0
# Absolute Basophils (10^3^/mcL)	0.05	0.01

**Table 2:** Summary of the patient's metabolic profile at initial presentation and after delirium. ALT: Alanine transaminase; AST: Aspartate transaminase.

Metabolic Panel	Initial Presentation	After Delirium
Glucose (mg/dl)	75	86
Na^+1^ (mEq/L)	138	137
K+1 (mEq/L)	3.9	4.5
Cl^-1 ^(mEq/L)	112	114
CO_2 _(mEq/L)	21	19
Ca^+2 ^(mEq/L)	8.0	7.9
BUN (mg/dl)	12	23
Creatinine (mg/dl)	0.8	0.7
Total Protein (g/dl)	6.9	5.0
Albumin (g/dl)	2.6	2.0
Bilirubin Total (mg/dl)	2.1	4.9
Bilirubin Direct (mg/dl)	0.9	2.0
Ammonia (umol/L)	Not measured	125
AST (U/L)	37	143
ALT (U/L)	23	139
Alk Phosphatase (U/L)	90.0	82.0
Osmolality (mOsm/L)	284.5	287.0

**Table 3:** Coagulation profile of the patient during initial presentation and after delirium. INR: International normalized ratio.

Coagulation Profile	Initial Presentation	After Delirium
Prothrombin Time (s)	14.0	14.5
INR	1.3	1.4
Partial Thromboplastin Time (s)	36	32

Treatment was targeted towards first identifying the cause of the acute changes in mental status. Brain computed tomography (CT) scan without contrast showed no abnormal findings. Dexamethasone was discontinued and the acute psychosis was treated with intramuscular injections of Haloperidol Lactate 2 mg and Lorazepam 2 mg prescribed by a hospital psychiatrist who evaluated the patient. After 48 hours of the onset of the acute changes in mental status, the patient regained orientation to time and place, was calm and cooperative, and able to follow commands. Hyperammonemia was treated with lactulose.

The patient was diagnosed with delirium induced by steroid therapy with precipitation of hepatic encephalopathy. On hospital day 7, he had foraminectomy and microdiscectomy lumbar spine surgery. He was transfused 20 units of platelets before surgery. The patient’s recovery after surgery was complicated leading to a prolonged hospital stay.

## Discussion

Despite the wide range of beneficial uses for perioperative steroids, they can cause psychiatric, endocrinologic, dermatologic, optic, gastrointestinal, and musculoskeletal adverse effects. In this case report, we describe the mental disturbance experienced by the patient after steroid therapy becoming disoriented and with poor attention hence identified as delirium. Warrington and Bostwick mentioned that the term "steroid-induced psychosis" is a way too simple explanation for describing complex psychiatric events [6]. Accordingly, our patient showed symptoms of delirium with disorientation to time, place and poor attention, and psychosis demonstrated by his poor reality testing and auditory hallucinations.

The patient met Diagnostic and Statistical Manual of Mental Disorders (DSM) V criteria for substance-induced psychotic disorder [7]. The symptoms observed developed after steroid therapy and resolved after 48 hours of its discontinuation. Furthermore, concomitant hyperammonemia and potentiation of steroid-induced psychosis due to impaired metabolism by a cirrhotic liver are the most likely explanations of the patients’ psychiatry disturbance. This exemplifies that there is currently a need for diagnostic laboratory tests to diagnose steroid-induced psychosis and the other substance-induced psychotic disorders. Thus, ruling out metabolic diseases that better explain a disturbance before making a diagnosis of substance-induced psychosis does not suffice in the clinical setting.

The venous total ammonia level correlates with the severity of hepatic encephalopathy, with ammonia levels between 50 umol/L and 100 umol/L correlating with hepatic encephalopathy measured at West Haven Criteria grade 2 [8]. Accordingly, the patient presented in this case had an ammonia level of 125 umol/L and symptoms of grade 2 hepatic encephalopathy during the disturbance. Lastly, at the time his psychiatry symptoms had resolved the ammonia level was normal. Thus, in this case, the onset and resolution of psychiatric symptoms correlate with ammonia levels.

The most common precipitating factors of hepatic encephalopathy in patients with liver disease are infection followed by constipation and gastrointestinal bleeding [9]. In addition, patients with two precipitating factors and advanced grade of HE have a prolonged hospital stay [10]. Benzodiazepines, narcotics, and alcohol are drugs that can precipitate HE. To the best of our knowledge, this is the first time of a reported case of a high dose of Dexamethasone therapy precipitating hepatic encephalopathy.

## Conclusions

This case provides a description of a patient with hepatic cirrhosis due to hepatitis C who became delirious after treatment with high doses of intravenous Dexamethasone. The clinical course was complicated due to the elevated ammonia level and symptomatology similar to that found in hepatic encephalopathy. Thus, diagnosing acute changes in mental status can be very challenging due to broad diagnoses including metabolic and psychiatric illnesses. Interestingly, we hypothesize the induction of delirium caused by potentiation of Dexamethasone in patients with hepatic cirrhosis due to impaired metabolism of the steroid. Furthermore, hepatic encephalopathy is another diagnosis that could not be excluded in the clinical setting of this patient, which was probably precipitated by steroid therapy. This case warrants further investigation about the undesired clinical and psychiatric manifestations of Dexamethasone and cautions physicians about the use of high dose of steroids in patients with hepatic cirrhosis.
